# Whole Genomic Analysis of an Unusual Human G6P[14] Rotavirus Strain Isolated from a Child with Diarrhea in Thailand: Evidence for Bovine-To-Human Interspecies Transmission and Reassortment Events

**DOI:** 10.1371/journal.pone.0139381

**Published:** 2015-09-30

**Authors:** Ratana Tacharoenmuang, Satoshi Komoto, Ratigorn Guntapong, Tomihiko Ide, Kei Haga, Kazuhiko Katayama, Takema Kato, Yuya Ouchi, Hiroki Kurahashi, Takao Tsuji, Somchai Sangkitporn, Koki Taniguchi

**Affiliations:** 1 Department of Medical Sciences, National Institute of Health, Nonthaburi, Thailand; 2 Department of Virology and Parasitology, Fujita Health University School of Medicine, Toyoake, Aichi, Japan; 3 Department of Virology II, National Institute of Infectious Diseases, Musashi-Murayama, Tokyo, Japan; 4 Division of Molecular Genetics, Institute for Comprehensive Medical Science, Fujita Health University, Toyoake, Aichi, Japan; 5 Genome and Transcriptome Analysis Center, Fujita Health University, Toyoake, Aichi, Japan; 6 Department of Microbiology, Fujita Health University School of Medicine, Toyoake, Aichi, Japan; The Pirbright Institute, UNITED KINGDOM

## Abstract

An unusual rotavirus strain, SKT-27, with the G6P[14] genotypes (RVA/Human-wt/THA/SKT-27/2012/G6P[14]), was identified in a stool specimen from a hospitalized child aged eight months with severe diarrhea. In this study, we sequenced and characterized the complete genome of strain SKT-27. On whole genomic analysis, strain SKT-27 was found to have a unique genotype constellation: G6-P[14]-I2-R2-C2-M2-A3-N2-T6-E2-H3. The non-G/P genotype constellation of this strain (I2-R2-C2-M2-A3-N2-T6-E2-H3) is commonly shared with rotavirus strains from artiodactyls such as cattle. Phylogenetic analysis indicated that nine of the 11 genes of strain SKT-27 (VP7, VP4, VP6, VP2-3, NSP1, NSP3-5) appeared to be of artiodactyl (likely bovine) origin, while the remaining VP1 and NSP2 genes were assumed to be of human origin. Thus, strain SKT-27 was found to have a bovine rotavirus genetic backbone, and thus is likely to be of bovine origin. Furthermore, strain SKT-27 appeared to be derived through interspecies transmission and reassortment events involving bovine and human rotavirus strains. Of note is that the VP7 gene of strain SKT-27 was located in G6 lineage-5 together with those of bovine rotavirus strains, away from the clusters comprising other G6P[14] strains in G6 lineages-2/6, suggesting the occurrence of independent bovine-to-human interspecies transmission events. To our knowledge, this is the first report on full genome-based characterization of human G6P[14] strains that have emerged in Southeast Asia. Our observations will provide important insights into the origin of G6P[14] strains, and into dynamic interactions between human and bovine rotavirus strains.

## Introduction

Group A rotavirus (RVA), a member of the *Reoviridae* family, is the most important etiological agent of severe gastroenteritis in the young of humans and many animal species worldwide. In humans, RVA infections are associated with high morbidity and mortality, being responsible for an estimated 453,000 deaths per year in children <5 years of age [1]. More than half of these deaths were estimated to occur in developing countries in Asia and Africa [1, 2].

The RVA virion is a triple-layered, non-enveloped icosahedron enclosing an 11-segment genome of double-stranded (ds)RNA [3]. RVA has two outer capsid proteins, VP7 and VP4, which are implicated independently in neutralization, and define the G and P genotypes, respectively. To date, RVA has been classified into at least 27 G and 37 P genotypes [4, 5]. Among them, some specific G and P genotypes are dominant in individual host species [6]. In human RVAs, 6 G genotypes (G1-4, G9, and G12) and 3 P genotypes (P[4], P[6], and P[8]) are commonly associated with human infections [7, 8]. In addition, several G genotypes (G5-6, G8, G10-11, and G20) and P genotypes (P[1]-[3], P[5], P[7], P[9]-[11], P[14], P[19], and P[25]) have been detected sporadically in humans [9–11]. Many of these unusual genotypes are believed to have originated from animal RVA strains that were introduced into the human population through interspecies transmission and/or reassortment events, one of the major ways in which the genetic diversity of RVAs is generated [6, 12, 13].

G6 strains, one of the most common G genotypes found in cattle [6, 13], have been identified in diarrheic children in combination with either a P[4], P[6], P[8], P[9], P[11], or P[14] genotype, most notably a P[14] genotype [14–17]. P[14] strains, the P genotype commonly found in rabbit and artiodactyls such as cattle, have also been found in children with diarrhea in combination with a G6 genotype [18]. The first G6P[14] strain, PA169, was identified in a child with acute gastroenteritis in Italy in 1988 [19], and subsequently in Australia [20–22], Belgium [18], Egypt [11], Hungary [23–25], India [26], Italy [15, 27], and Spain [28]. In host species other than humans, G6P[14] strains have been detected only in artiodactyls (antelope and goat) in South Africa [18]. In 2012, we detected the first Thai G6 strain from a diarrheic child in a total of 685 RVA-positive stool specimens by PCR-based genotyping (Tacharoenmuang et al., in preparation).

A whole genome-based genotyping system was recently proposed for RVAs based on assignment of all the 11 gene segments (i.e., G/P and non-G/P genotypes) [29]. In the new genotyping system, the acronym Gx-P[x]-Ix-Rx-Cx-Mx-Ax-Nx-Tx-Ex-Hx, where x is an integer, defines the genotype of the VP7-VP4-VP6-VP1-VP2-VP3-NSP1-NSP2-NSP3-NSP4-NSP5 genes, respectively, of a given RVA strain. Most human RVA strains have genes similar in sequence to those of prototype human strains Wa (RVA/Human-tc/USA/Wa/1974/G1P[8]), DS-1 (RVA/Human-tc/USA/DS-1/1976/G2P[4]), or AU-1 (RVA/Human-tc/JPN/AU-1/1982/G3P[9]) [30]. The Wa-like strains are characterized by non-G/P genotypes (I1-R1-C1-M1-A1-N1-T1-E1-H1), and tend to have G/P genotypes G1P[8], G3P[8], G4P[8], and G9P[8]. In contrast, the DS-1-like strains are characterized by non-G/P genotypes (I2-R2-C2-M2-A2-N2-T2-E2-H2), and tend to have G/P genotype G2P[4]. The third minor AU-1-like strains are characterized by non-G/P genotypes (I3-R3-C3-M3-A3-N3-T3-E3-H3), and tend to have G/P genotype G3P[9]. Whole genome-based analysis is a reliable method for obtaining conclusive data on the origin of an RVA strain, and for tracing its evolutionary pattern [29, 31]. To date, however, the whole genomes of some G6P[14] strains from Europe, Australia, and Africa have been analyzed, which provided evidence of their artiodactyl (likely bovine) origin [18, 25, 29]. G6P[14] strains are speculated to be the result of reassortment between strains from cattle and other member(s) of the Artiodactyla [18]. Partial-length genomic analyses of G6P[14] strains from Africa and Asia have also shown the presence of a unique non-G/P genotype constellation (I2-R2-C2-M2-(A3/A11)-N2-T6-E2-H3), commonly shared with RVA strains from artiodactyls including cattle [17, 26]. However, the overall genomic constellation and the exact evolutionary patterns of G6P[14] strains remain to be elucidated. Although only one Asian G6P[14] strain, Indian human N-1, has been studied, its genomic analysis was based on partial-length genome sequences. Thus, we analyzed the whole genome of Thai strain SKT-27 with the G6P[14] genotypes, to gain a more precise understanding of the evolutionary patterns of G6P[14] strains in Asia. In the present study, we analyzed the whole genome of the first Thai strain, SKT-27, with the G6P[14] genotypes isolated from a diarrheic child. Moreover, in this study, deep sequencing with the next generation sequencing (NGS) Illumina MiSeq platform was carried out to obtain the complete nucleotide sequence of the whole genome of strain SKT-27.

## Materials and Methods

### Ethics statement

The study was approved by the Ethical Review Committee for Research in Human Subjects of the Ministry of Public Health, Thailand (Ref. no. 10/2555). In this study, written informed consent for the testing of stool samples for RVAs and characterization of identified RVA strains was obtained from the children’s parents/guardians.

### Virus strains

The full-genomic sequence was determined for strain SKT-27, which was identified as the sole pathogen causing diarrhea in stool specimens from a hospitalized child aged eight months with severe diarrhea in Sukhothai Province in November 2012 during the RVA strain surveillance program in Phetchaboon and Sukhothai Provinces, Thailand in 2012–2014, which involved a total of 685 RVA-positive fecal samples (Tacharoenmuang et al., in preparation). Stool specimens were collected from hospital-based surveillance activities under the RVA vaccine effectiveness evaluation study in Thailand. Stool samples containing strain SKT-27 were kept at −30°C until use.

### Viral dsRNA extraction

The viral dsRNAs were extracted from stool suspensions using a QIAamp Viral RNA Mini Kit (Qiagen). The extracted dsRNAs were used for (i) polyacrylamide gel electrophoresis (PAGE) analysis, and (ii) whole genomic analysis. For PAGE analysis, the dsRNAs were electrophoresed in a 10% polyacrylamide gel for 16 h at 20 mA at room temperature, followed by silver staining [32] to determine the genomic dsRNA profile. For whole genomic analysis, viral dsRNAs were subjected to Illumina MiSeq sequencing as described below.

### cDNA library building and Illumina MiSeq sequencing

Preparation of a cDNA library and Illumina MiSeq sequencing were carried out as described previously [30, 33]. Briefly, a 200 bp fragment library ligated with bar-coded adapters was constructed for strain SKT-27 using an NEBNext Ultra RNA Library Prep Kit for Illumina v1.2 (New England Biolabs) according to the manufacturer’s instructions. Library purification was performed using Agencourt AMPure XP magnetic beads (Beckman Coulter). The quality of the purified cDNA library was assessed on an Agilent 2100 Bioanalyzer (Agilent Technologies). Nucleotide sequencing was performed on an Illumina MiSeq sequencer (Illumina) using a MiSeq Reagent Kit v2 (Illumina) to generate 151 paired-end reads. Data analysis was carried out using CLC Genomics Workbench v8.0.1 (CLC Bio). Contigs were assembled from the obtained sequence reads by *de novo* assembly. Using the assembled contigs as query sequences, the Basic Local Alignment Search Tool (BLAST) non-redundant nucleotide database was searched to obtain the full-length nucleotide sequence of each gene segment of strain SKT-27. The nucleotide sequences were translated into amino acid sequences using GENETYX v11 (GENETYX).

### Determination of RVA genotypes

The genotype of each of the 11 gene segments of strain SKT-27 was determined using the RotaC v2.0 automated genotyping tool (http://rotac.regatools.be/) [34] according to the guidelines proposed by the Rotavirus Classification Working Group (RCWG).

### Phylogenetic analyses

Sequence comparisons were performed as described previously [33, 35]. Briefly, multiple alignment of each viral gene was performed using CLUSTAL W [36]. Phylogenetic trees were constructed using the maximum likelihood method and the Tamura-Nei substitution model using MEGA6.06 [37]. The reliability of the branching order was estimated from 1000 bootstrap replicates [38]. The results of phylogenetic analyses were validated using several other genetic distance models, such as the Jukes-Cantor, Kimura 2-parameter, Tamura 3-parameter, and Hasegawa-Kishino-Yano ones (data not shown).

### Nucleotide sequence accession numbers

The nucleotide sequence data presented in this manuscript have been deposited in the DDBJ and EMBL/GenBank data libraries. The accession numbers for the nucleotide sequences of the VP1-4, VP6-7, and NSP1-5/6 genes of strain SKT-27 are LC055547-LC055557, respectively.

## Results and Discussion

### Extraction of genomic dsRNAs of strain SKT-27

**Fig 1:**
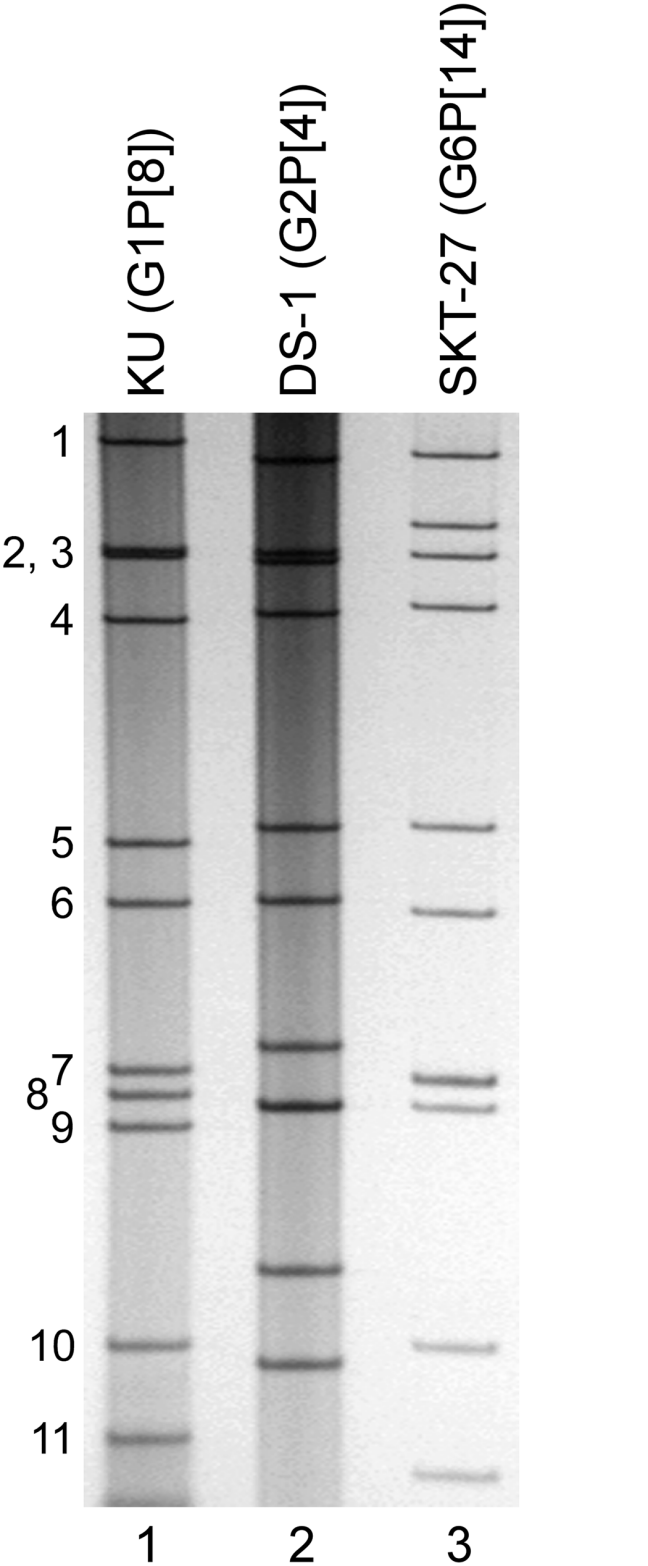
Genomic dsRNA profile of strain SKT-27. Lanes 1–2, dsRNAs of strains KU (G1P[8]) (lane 1) and DS-1 (G2P[4]) (lane 2), respectively, extracted from the cell cultures; lane 3, dsRNAs of strain SKT-27 extracted from a stool sample. The numbers on the left indicate the order of the genomic dsRNA segments of strain KU.

Virion dsRNAs of strain SKT-27 were extracted from stool specimens and then analyzed by PAGE. Fig 1 shows the profiles of viral dsRNAs from human strains KU (RVA/Human-tc/JPN/KU/1974/G1P[8]) [39] (lane 1) and DS-1 (G2P[4]) [40] (lane 2), as references, extracted from the cell cultures, and strain SKT-27 (lane 3) from a stool sample. Strain SKT-27 showed a long electropherotype.

### Nucleotide sequencing and whole-genome-based genotyping of strain SKT-27

PCR-based genotyping [41] showed that strain SKT-27 has the G6 genotype (Tacharoenmuang et al., in preparation). Because G6 strains have been detected almost exclusively in artiodactyls such as cattle [6, 18], the detection of a G6 strain in the human population was suggestive of zoonotic transmission. The whole genome of strain SKT-27 was amplified using a sequence-independent primer set and then sequenced successfully. Illumina MiSeq sequencing yielded 6.8 x 10^5^ reads (~145 bp average length) for strain SKT-27. Complete or nearly complete nucleotide sequences of all the 11 gene segments of strain SKT-27 could be obtained.

**Fig 2:**
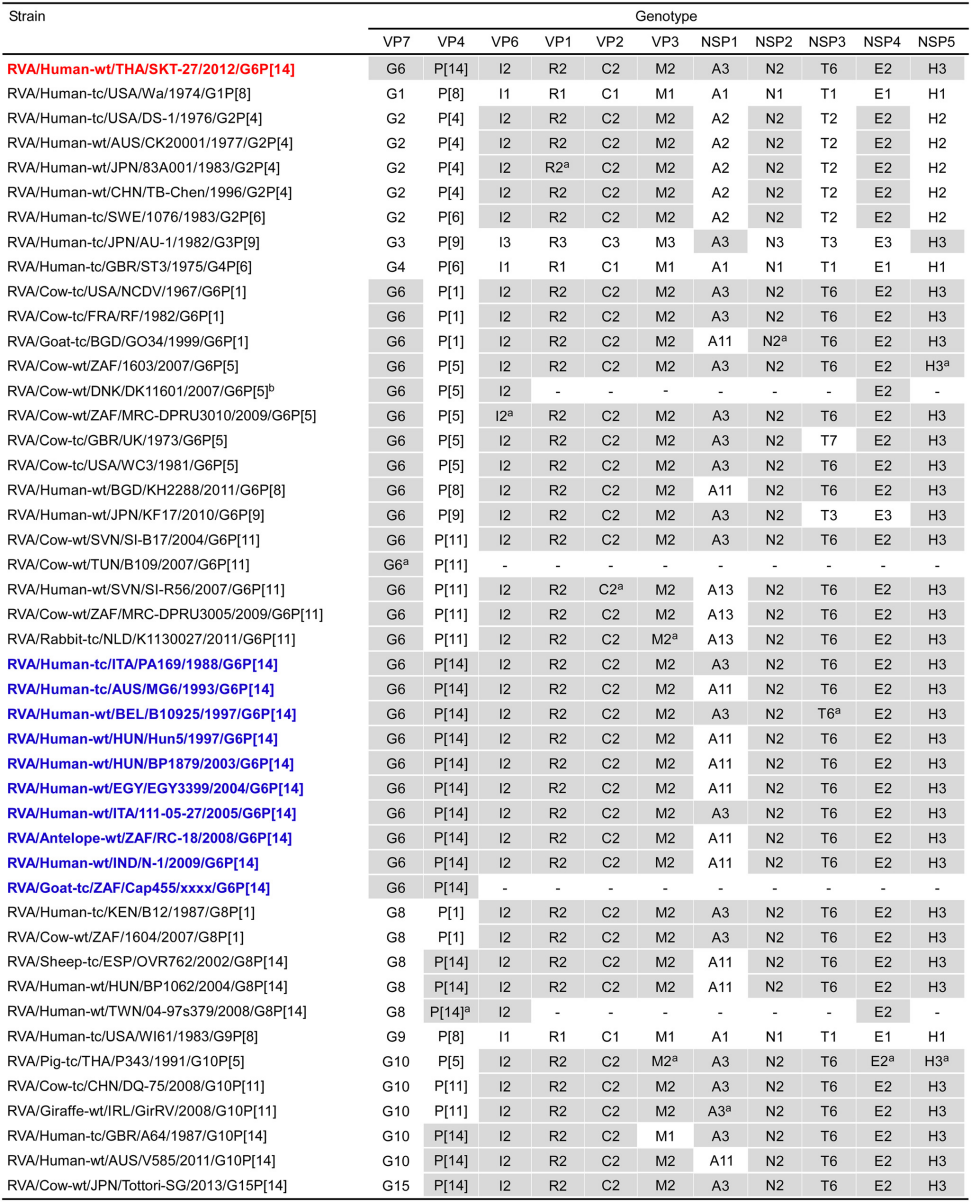
Genotype natures of the 11 gene segments of strain SKT-27 compared with those of selected human and animal strains. Strain SKT-27 is shown in red, while other G6P[14] strains are shown in blue. Gray shading indicates the gene segments with a genotype identical to that of strain SKT-27. ^a^The gene segments that are most similar to those of strain SKT-27. “−” indicates that no sequence data were available in the DDBJ and EMBL/GenBank data libraries. ^b^Genotype assignment of strain DK11601 based on a report by Midgley et al. (2012). To our knowledge, to date, nucleotide sequence accession numbers for the VP7 and VP4 genes of strain DK11601 are not available in the DDBJ and EMBL/GenBank data libraries.

The 11 genes of strain SKT-27 were assigned as G6-P[14]-I2-R2-C2-M2-A3-N2-T6-E2-H3 (Fig 2). Strain SKT-27 was confirmed to have the G6 genotype as determined by PCR-based genotyping. Furthermore, the P genotype of strain SKT-27 was successfully determined to be P[14] using the Illumina MiSeq sequencing approach, although the VP4 gene of this strain could not be genotyped by PCR-based genotyping (Tacharoenmuang et al., in preparation). Thus, strain SKT-27 was named RVA/Human-wt/THA/SKT-27/2012/G6P[14] according to the guidelines for the uniformity of RVAs proposed by the RCWG. Comparison of the complete genotype constellation of strain SKT-27 with those of other G6 and non-G6 strains is shown in Fig 2. Strain SKT-27 exhibited a unique non-G/P genotype constellation (I2-R2-C2-M2-A3-N2-T6-E2-H3), which is commonly found in RVA strains from artiodactyls such as cattle [17, 18, 42]. Strain SKT-27 was found to share the same non-G/P genotype constellation with bovine strains (NCDV (RVA/Cow-tc/USA/NCDV/1967/G6P[1]), RF (RVA/Cow-tc/FRA/RF/1982/G6P[1]), 1603 (G6P[5]), MRC-DPRU3010 (G6P[5]), WC3 (RVA/Cow-tc/USA/WC3/1981/G6P[5]), SI-B17 (G6P[11]), 1604 (G8P[1]), DQ-75 (G10P[11]), and Tottori-SG (G15P[14])), bovine-like human strains (B12 (G8P[1]), PA169 (G6P[14]), B10925 (G6P[14]), and 111-05-27 (G6P[14])), bovine-like porcine strain P343 (G10P[5]), and bovine-like giraffe strain GirRV (G10P[11]), although some bovine and bovine-like strains have been found to have other NSP1 (A11 or A13 instead of A3) and NSP3 (T3 or T7 instead of T6) genotypes. Human strains (B12, PA169, B10925, and 111-05-27), porcine strain P343, and giraffe strain GirRV have been shown to have bovine backbones, and to be likely of bovine origin through their full genomic analyses [12, 29, 43, 44]. Thus, the genotype constellation of strain SKT-27 is mostly identical to those of bovine and bovine-like strains.

### Phylogenetic analyses

We next constructed phylogenetic trees using the full-genome sequence for each of the 11 gene segments because phylogenetic analysis of RVA nucleotide sequences provides direct evidence of their relatedness to those of other strains, even within the same genotype [29].

**Fig 3:**
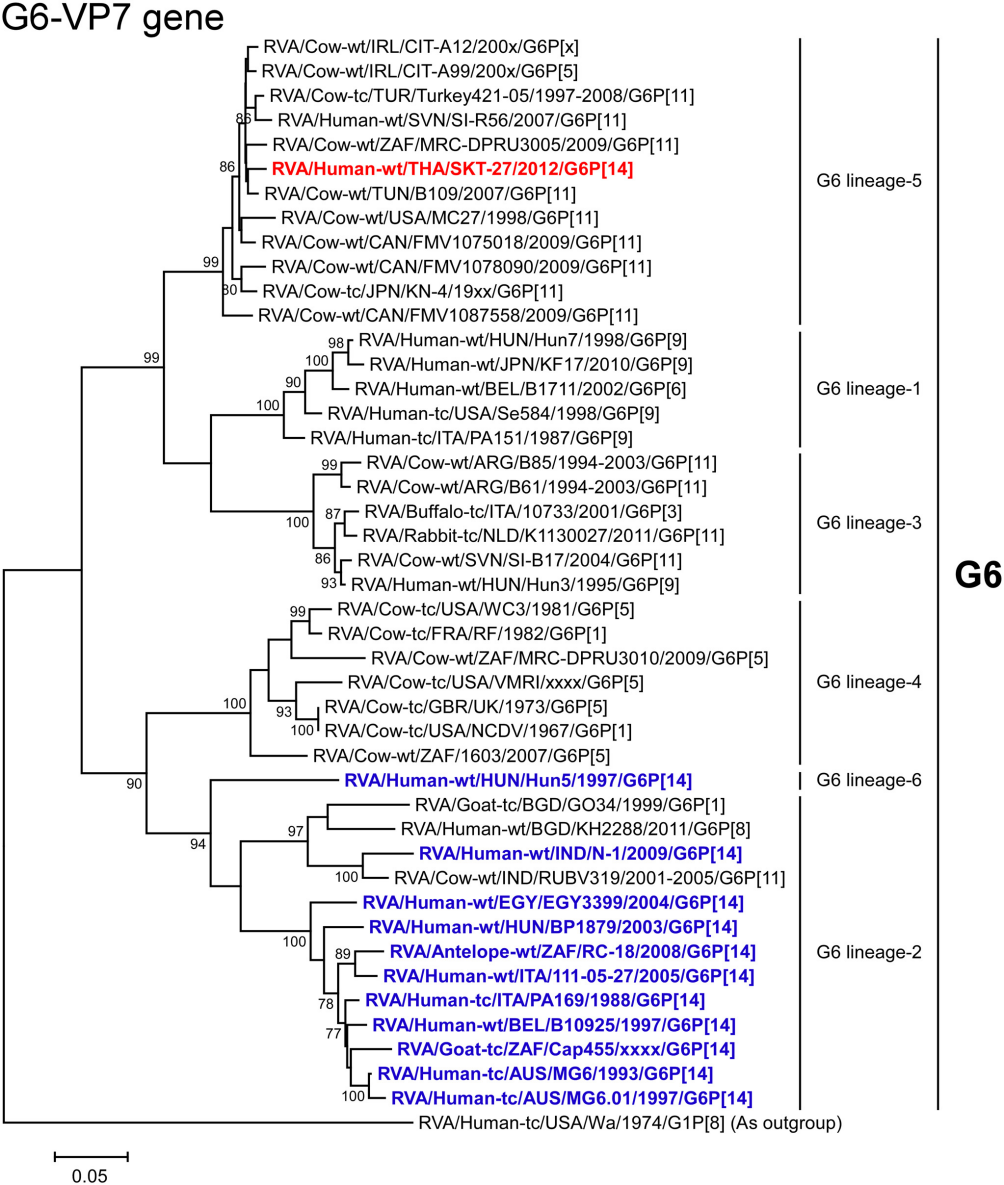
Phylogenetic tree constructed from the nucleotide sequences of the VP7 genes of strain SKT-27 and representative RVA strains. In the tree, the position of strain SKT-27 is shown in red, while other G6P[14] strains are shown in blue. Bootstrap values of <75% are not shown. Scale bars: 0.05 substitutions per nucleotide.

The VP7 gene of strain SKT-27 exhibited the maximum nucleotide sequence identity (98.4%) with that of Tunisian bovine strain B109 (G6P[11]) [45] (Fig 2), and comparable identities (98.1–98.2%) with Irish bovine strains CIT-A12 (G6P[x]) and CIT-A99 (G6P[5]) [46]. On phylogenetic analysis, strain SKT-27 was found to form a cluster with the above-mentioned bovine strains, and several bovine and bovine-like strains with G6P[11] genotypes in G6 lineage-5, away from the clusters comprising other G6P[14] strains in G6 lineages-2/6 (Fig 3).

**Fig 4:**
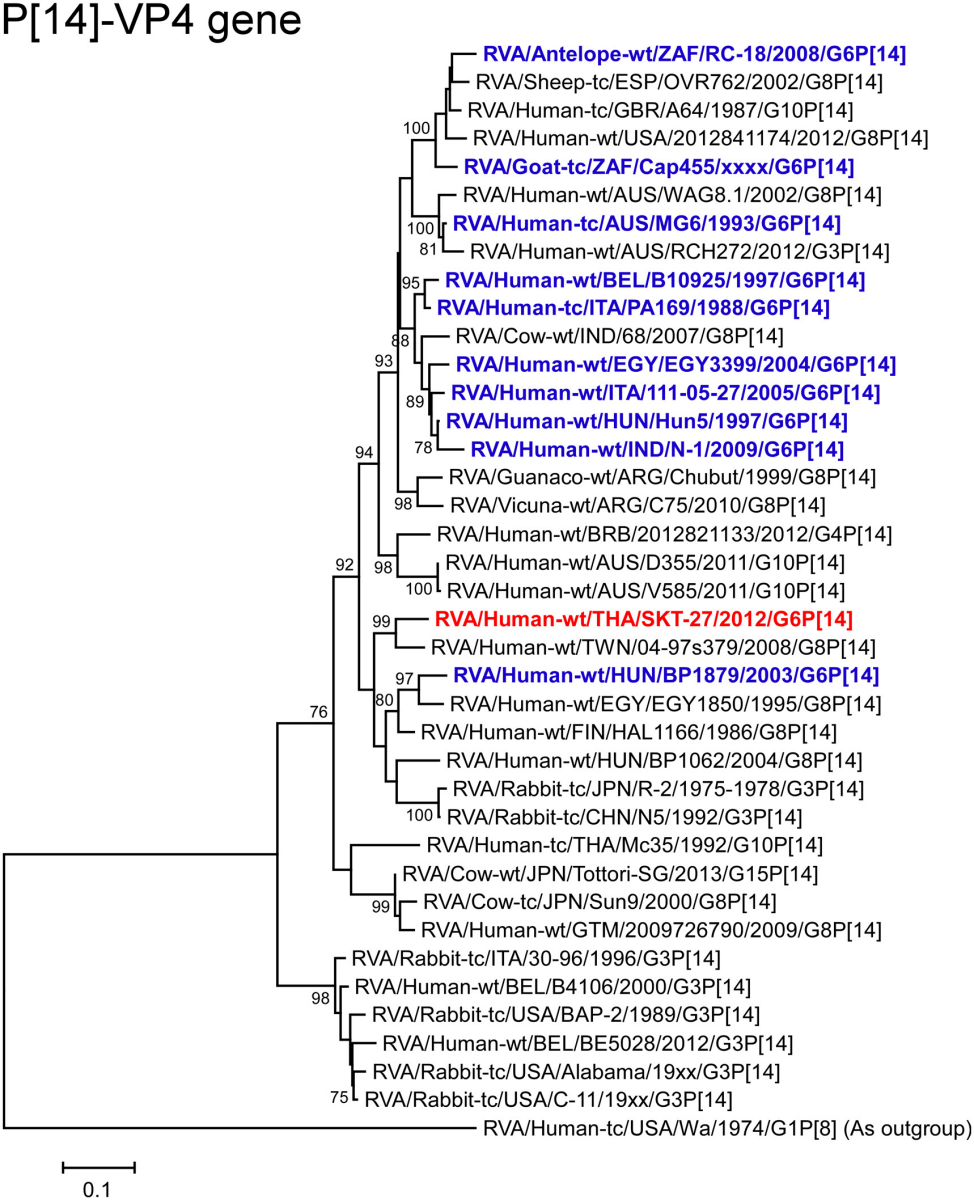
Phylogenetic tree constructed from the nucleotide sequences of the VP4 genes of strain SKT-27 and representative RVA strains. See legend of Fig 3. Scale bars: 0.1 substitutions per nucleotide.

The VP4 gene of strain SKT-27 showed the highest nucleotide sequence identity (92.4%) with the cognate gene of Taiwanese bovine-like human strain 04-97s379 (G8P[14]) [47] (Fig 2), and somewhat lower identity (90.0%) with Finnish bovine-like human strain HAL1166 (G8P[14]) [48–50]. On phylogenetic analysis, strain SKT-27 was shown to be closely related with strain 04-97s379 in a common branch with strain HAL1166 (Fig 4).

**Fig 5:**
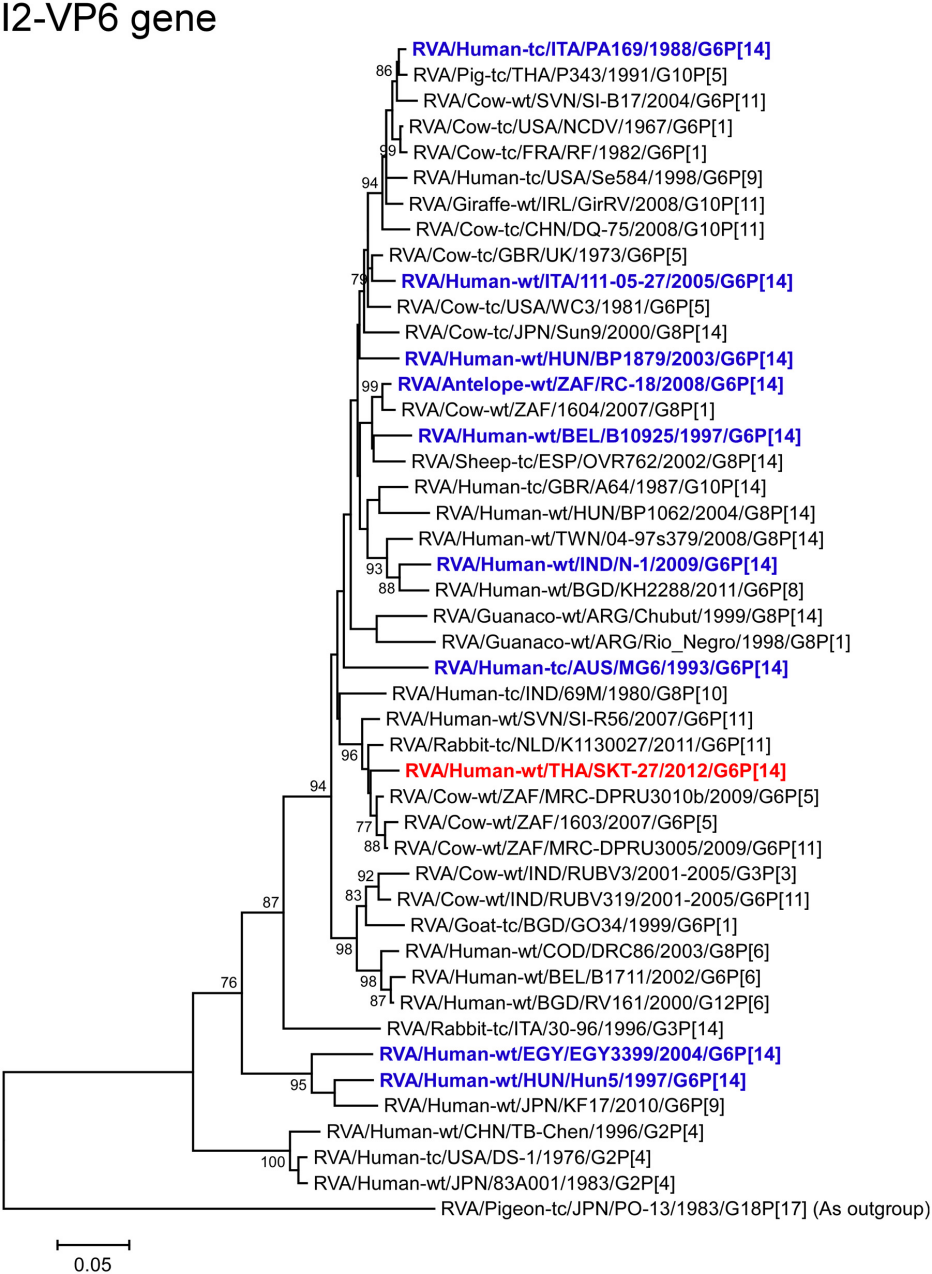
Phylogenetic tree constructed from the nucleotide sequences of the VP6 genes of strain SKT-27 and representative RVA strains. See legend of Fig 3. Scale bars: 0.05 substitutions per nucleotide.

The VP6 gene of strain SKT-27 exhibited the highest nucleotide sequence identity (97.8%) with the VP6 gene of South African bovine strain MRC-DPRU3010b (G6P[5]) [51] (Fig 2), and comparable identities (97.5–97.7%) with South African bovine strains 1603 (G6P[5]) [52] and MRC-DPRU3005 (G6P[11]). Phylogenetically, strain SKT-27 was closely related with these South African bovine strains in a common branch with Dutch bovine-like lapine strain K1130027 (G6P[11]) [53] (Fig 5).

**Fig 6:**
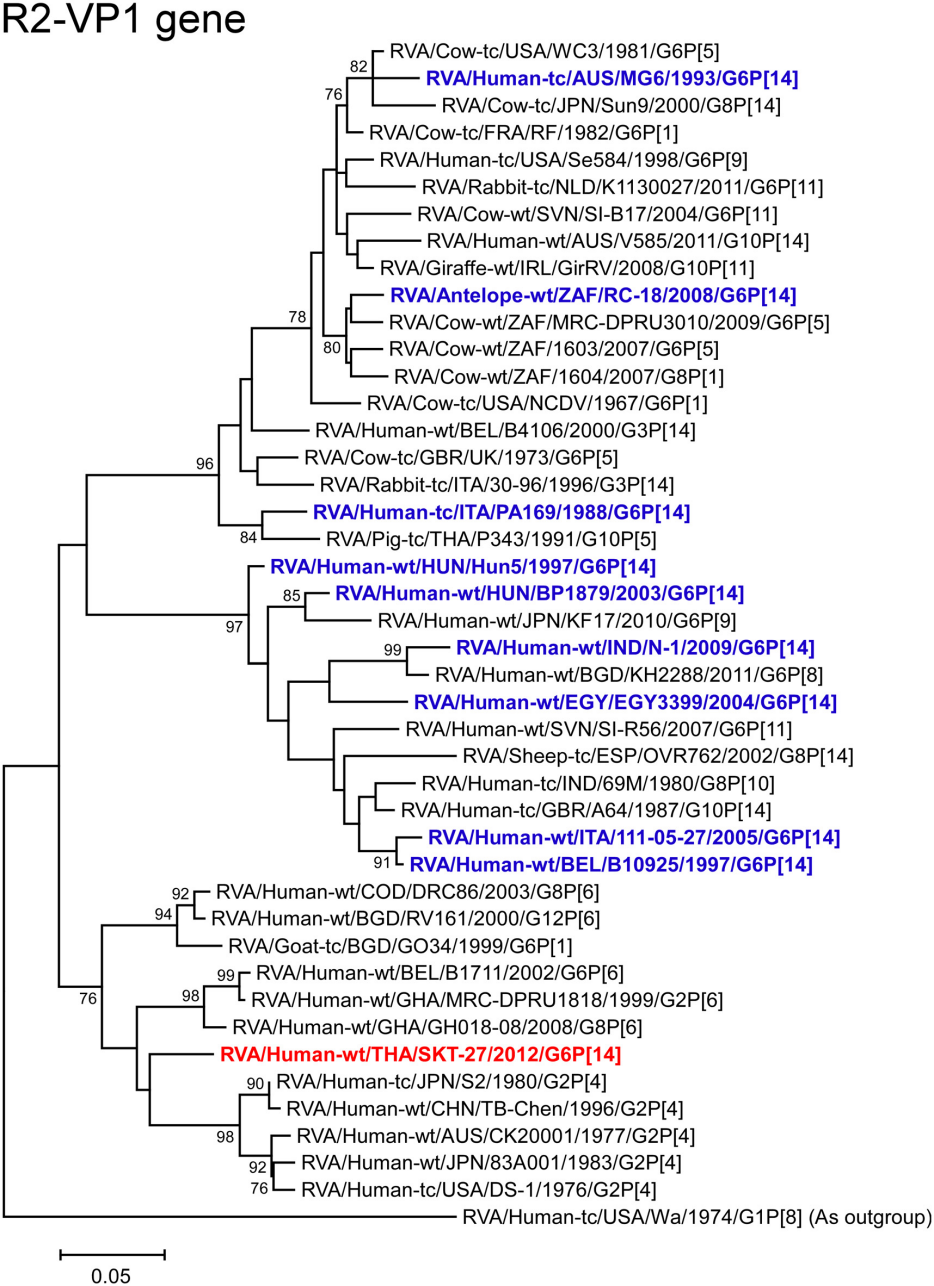
Phylogenetic tree constructed from the nucleotide sequences of the VP1 genes of strain SKT-27 and representative RVA strains. See legend of Fig 3. Scale bars: 0.05 substitutions per nucleotide.

The VP1 gene of strain SKT-27 showed the maximum nucleotide sequence identity (92.7%) with the cognate gene of Japanese human strain 83A001 (G2P[4]) [54] (Fig 2), and comparable identities (92.3–92.6%) with reference human strains DS-1 (G2P[4]), S2 (RVA/Human-tc/JPN/S2/1980/G2P[4]), and TB-Chen (RVA/Human-wt/CHN/TB-Chen/1996/G2P[4]) [40, 55, 56]. On phylogenetic analysis, strain SKT-27 was shown to cluster near the prototype human G2P[4] strains DS-1 and S2 (Fig 6).

**Fig 7:**
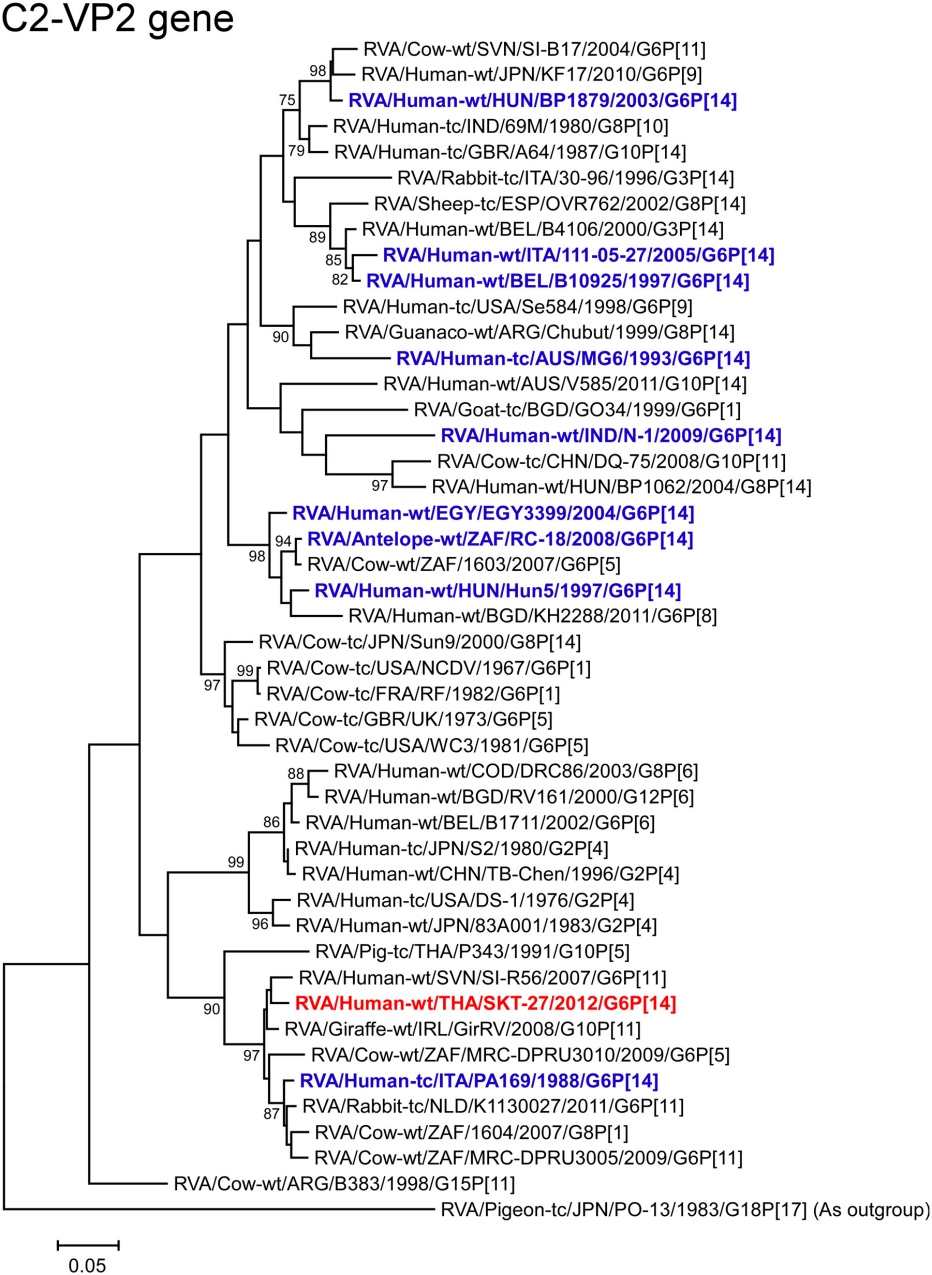
Phylogenetic tree constructed from the nucleotide sequences of the VP2 genes of strain SKT-27 and representative RVA strains. See legend of Fig 3. Scale bars: 0.05 substitutions per nucleotide.

The VP2 gene of strain SKT-27 showed the highest nucleotide sequence similarity (96.7%) with the VP2 gene of Slovene bovine-like human strain SI-R56 (G6P[11]) [57] (Fig 2), and comparable identity (96.5%) with Irish bovine-like giraffe strain GirRV (G10P[11]) [44]. Phylogenetically, strain SKT-27 was shown to be closely related with strain SI-R56 in a common branch with strain GirRV (Fig 7).

**Fig 8:**
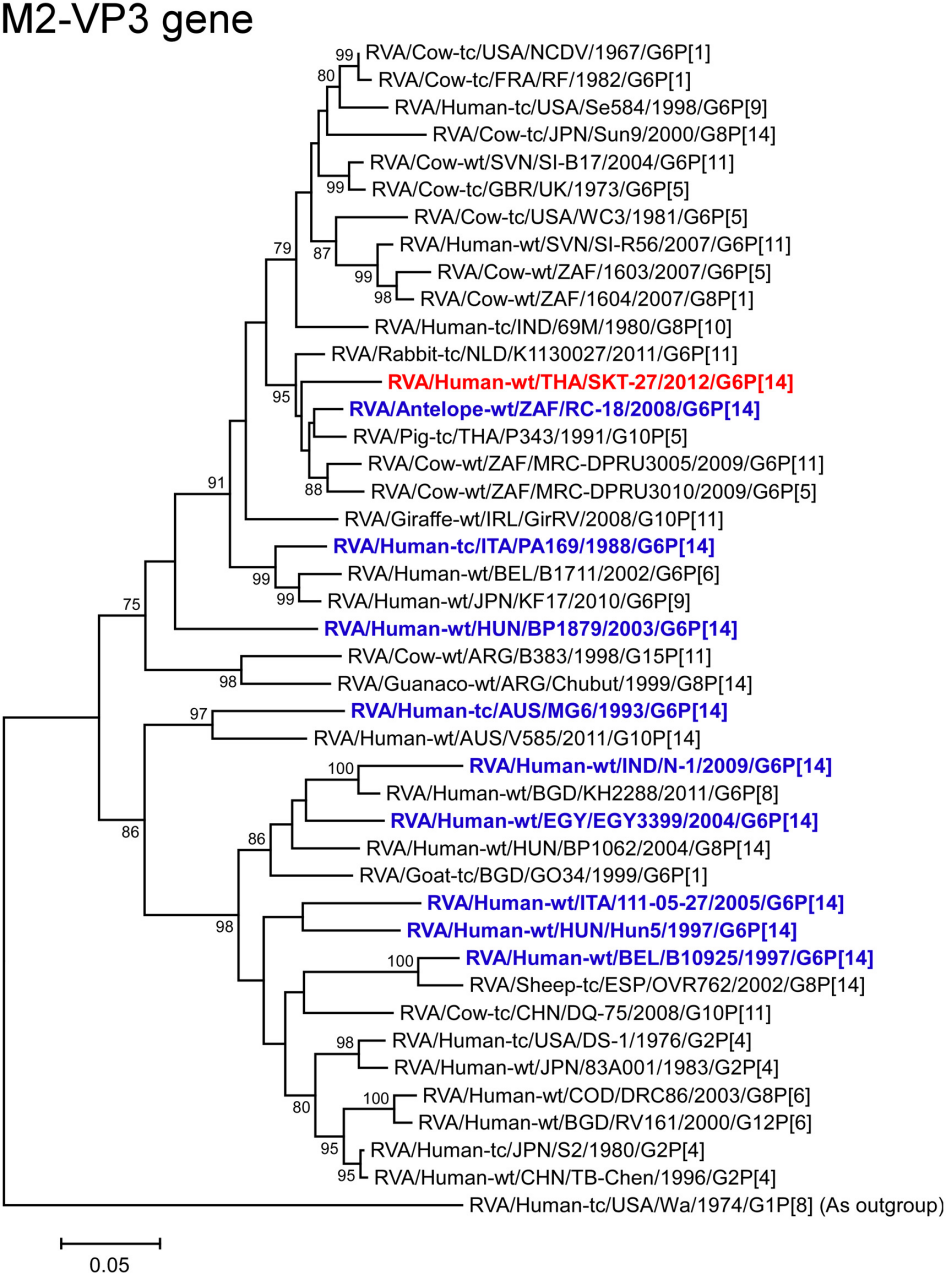
Phylogenetic tree constructed from the nucleotide sequences of the VP3 genes of strain SKT-27 and representative RVA strains. See legend of Fig 3. Scale bars: 0.05 substitutions per nucleotide.

The VP3 gene of strain SKT-27 showed the maximum nucleotide sequence identity (95.4%) with those of Thai bovine-like porcine strain P343 (G10P[5]) [43] and Dutch bovine-like lapine strain K1130027 (G6P[11]) (Fig 2), and comparable similarity (95.3%) with South African bovine-like antelope strain RC-18 (G6P[14]) [18]. On phylogenetic analysis, strain SKT-27 was closely related with these bovine-like strains, and South African bovine strains MRC-DPRU3010 (G6P[5]) and MRC-DPRU3005 (G6P[11]) (Fig 8).

**Fig 9:**
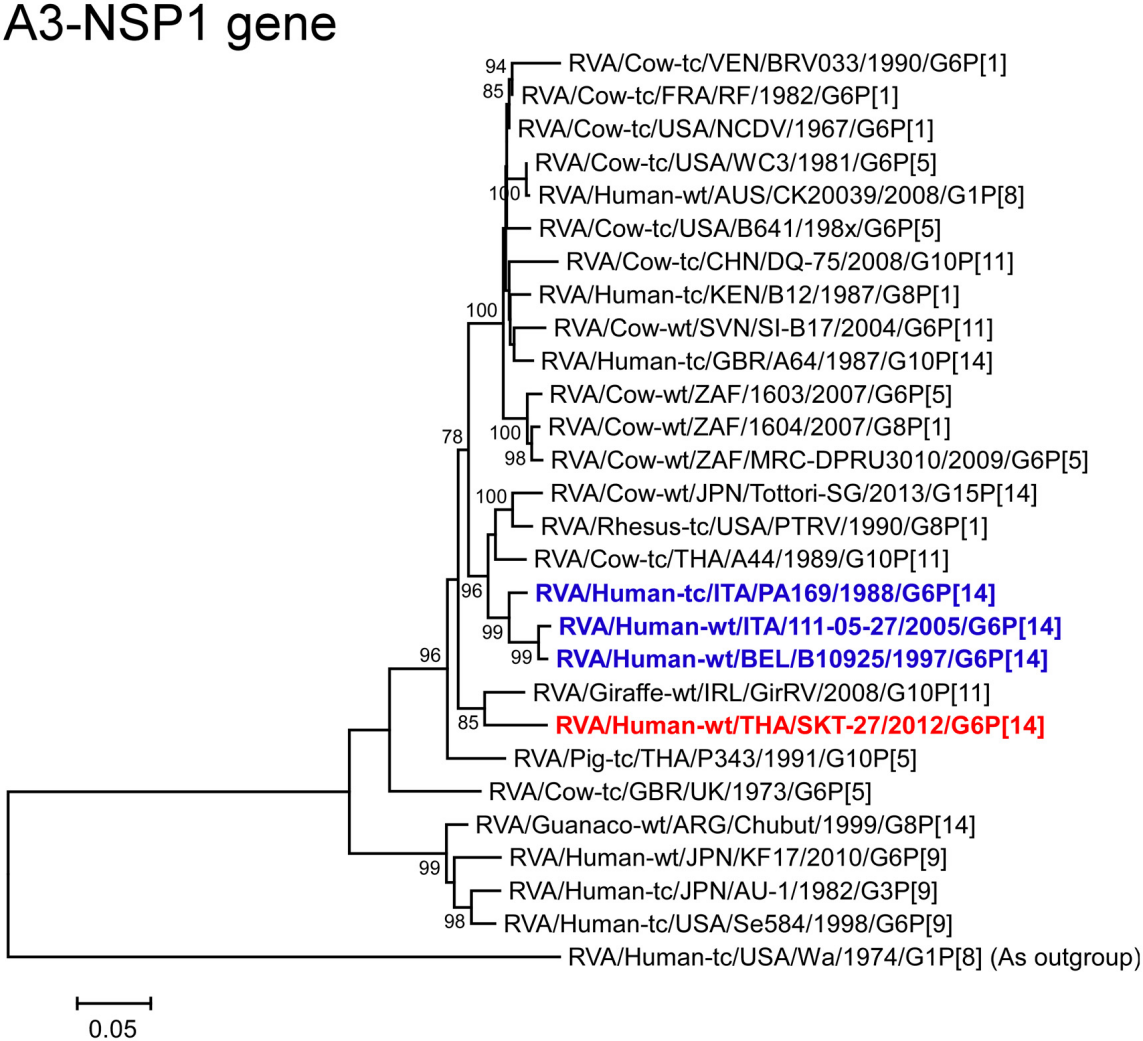
Phylogenetic tree constructed from the nucleotide sequences of the NSP1 genes of strain SKT-27 and representative RVA strains. See legend of Fig 3. Scale bars: 0.05 substitutions per nucleotide.

The NSP1 gene of strain SKT-27 exhibited the highest nucleotide sequence identity (93.7%) with the cognate gene of Irish bovine-like giraffe strain GirRV (G10P[11]) (Fig 2). On phylogenetic analysis, strain SKT-27 was shown to be closely related with strain GirRV near the clusters formed by several bovine and bovine-like strains (Fig 9).

**Fig 10:**
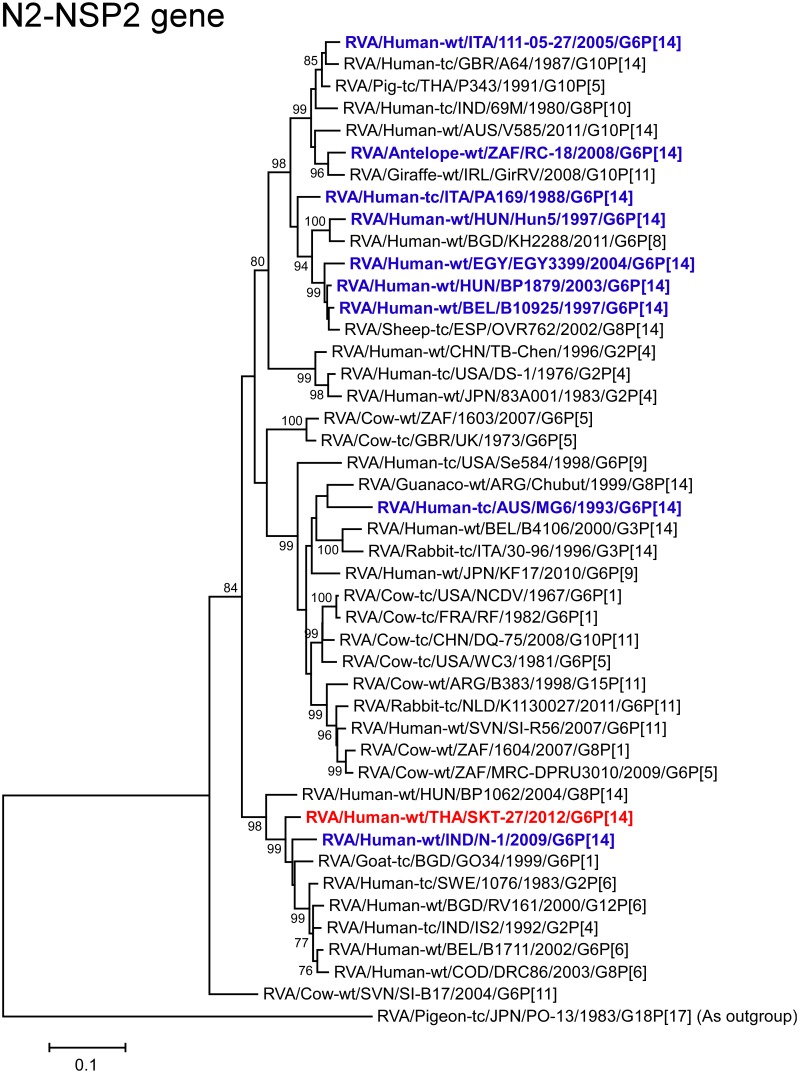
Phylogenetic tree constructed from the nucleotide sequences of the NSP2 genes of strain SKT-27 and representative RVA strains. See legend of Fig 3. Scale bars: 0.1 substitutions per nucleotide.

The NSP2 gene of strain SKT-27 exhibited the maximum nucleotide sequence identity (95.4%) with that of caprine strain GO34 (RVA/Goat-tc/BGD/GO34/1999/G6P[1]) from Bangladesh [58] (Fig 2), and comparable identity (95.3%) with Swedish human strain 1076 (G2P[6]) [59]. Phylogenetically, strain SKT-27 was found to cluster near these, and several human G2, G6, G8, and G12 strains from different parts of the world (Fig 10).

**Fig 11:**
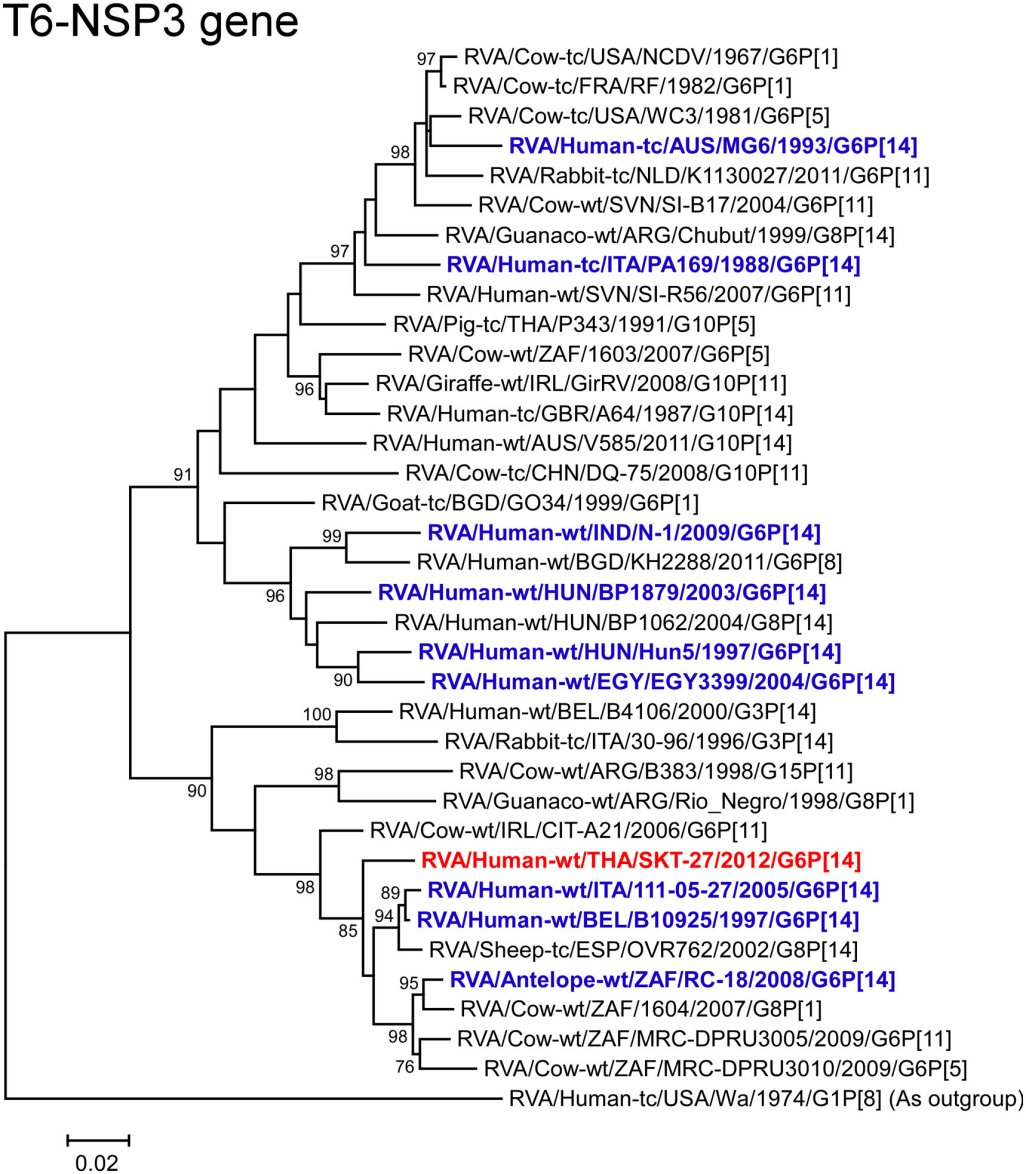
Phylogenetic tree constructed from the nucleotide sequences of the NSP3 genes of strain SKT-27 and representative RVA strains. See legend of Fig 3. Scale bars: 0.02 substitutions per nucleotide.

The NSP3 gene of strain SKT-27 showed the highest nucleotide sequence identity (97.2%) with the NSP3 gene of Belgian bovine-like strain human B10925 (G6P[14]) [18] (Fig 2), and somewhat lower identities (96.6–96.8%) with Italian bovine-like human strain 111-05-27 (G6P[14]) [18] and Spanish bovine-like ovine strain OVR762 (G8P[14]) [18]. On phylogenetic analysis, strain SKT-27 was shown to cluster near these bovine-like P[14] strains (Fig 11).

**Fig 12:**
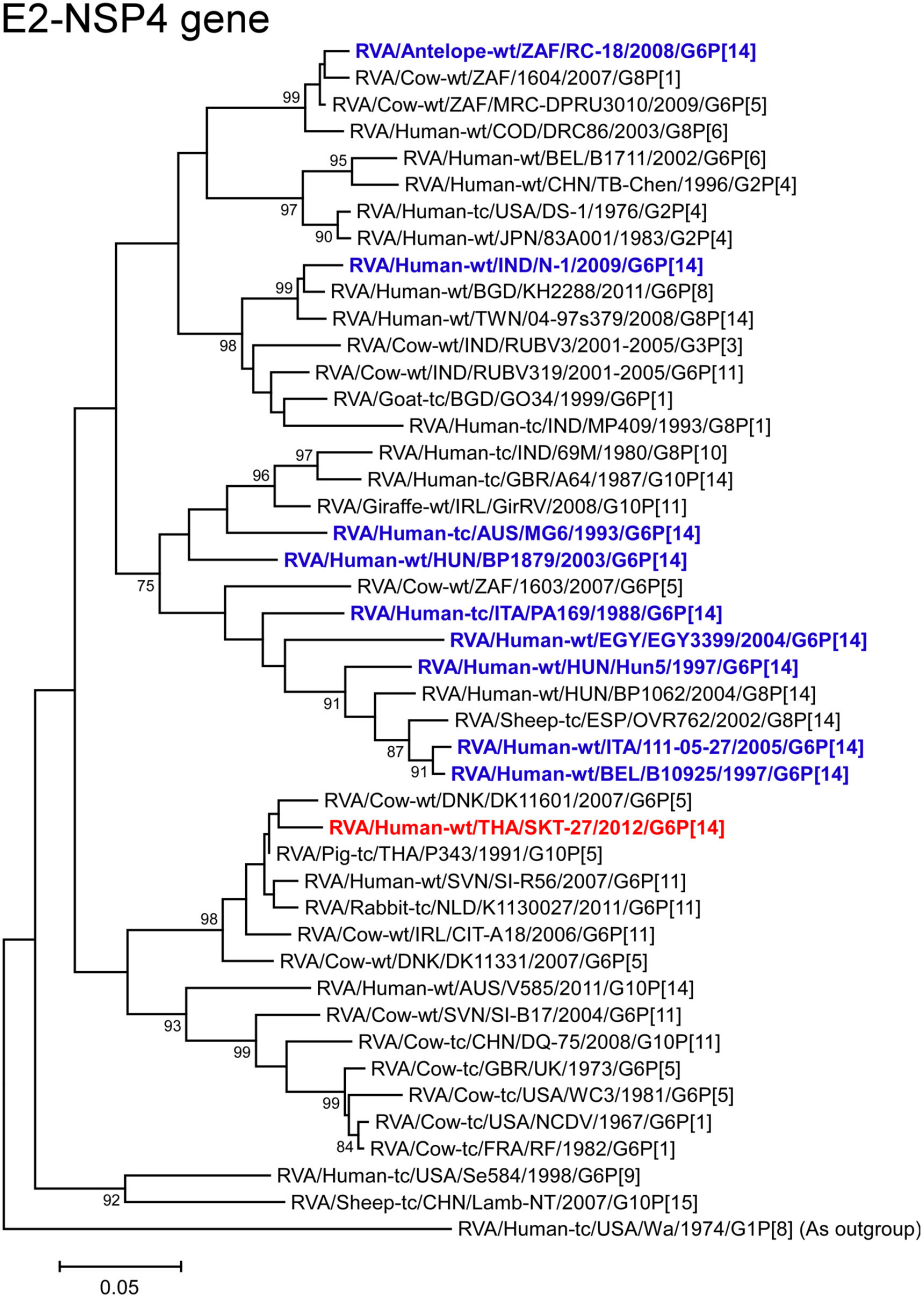
Phylogenetic tree constructed from the nucleotide sequences of the NSP4 genes of strain SKT-27 and representative RVA strains. See legend of Fig 3. Scale bars: 0.05 substitutions per nucleotide.

The NSP4 gene of strain SKT-27 showed the maximum nucleotide sequence similarity (98.5%) with the cognate gene of Thai bovine-like porcine strain P343 (G10P[5]) (Fig 2), and somewhat lower identity (97.4%) with Danish bovine strain DK11601 (G6P[5]) [60]. On phylogenetic analysis, strain SKT-27 was found to be closely related with strain DK11601 in a common branch with strain P343, Slovene bovine-like human strain SI-R56 (G6P[11]), and Dutch bovine-like lapine strain K1130027 (G6P[11]) (Fig 12).

**Fig 13:**
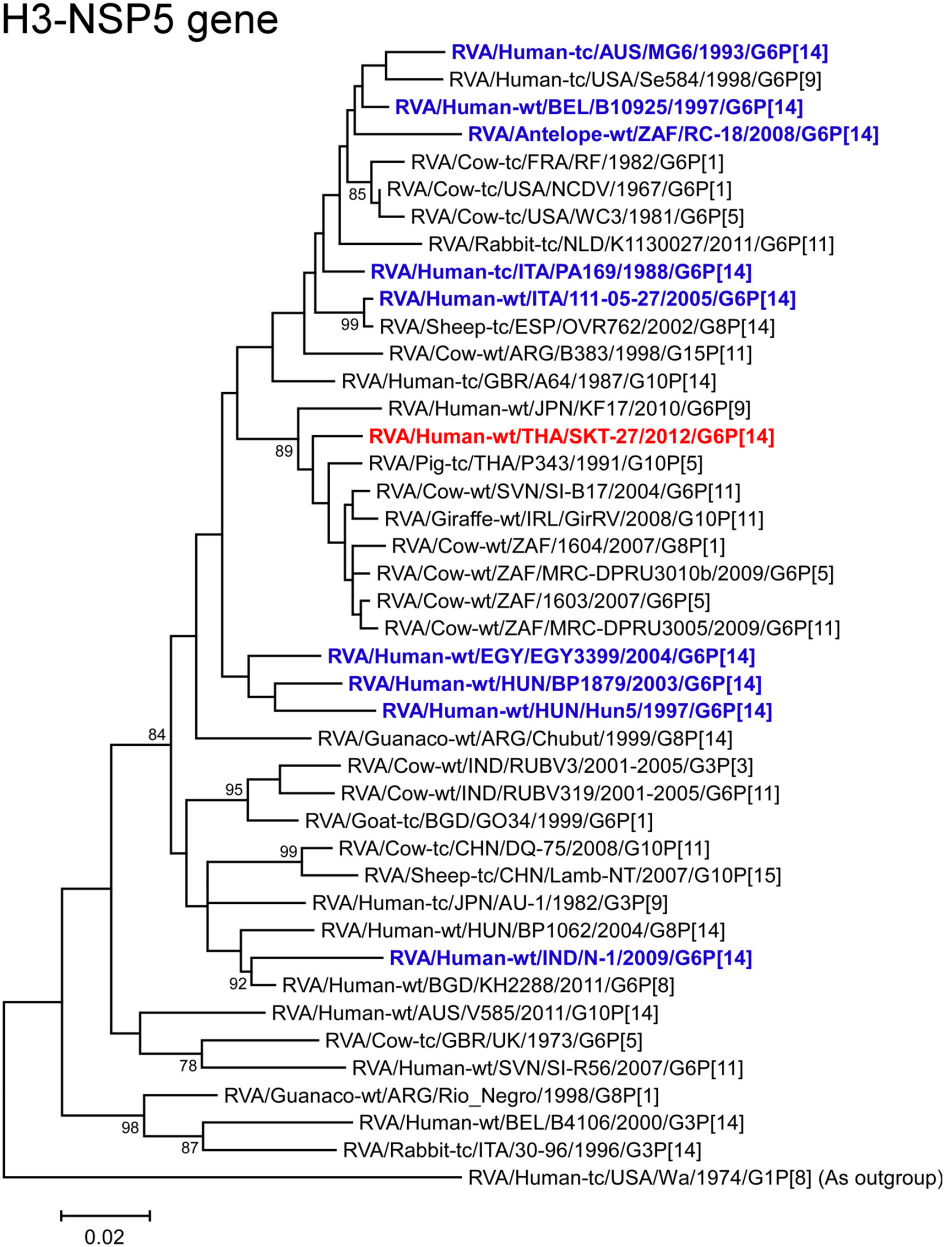
Phylogenetic tree constructed from the nucleotide sequences of the NSP5 genes of strain SKT-27 and representative RVA strains. See legend of Fig 3. Scale bars: 0.02 substitutions per nucleotide.

The NSP5 gene of strain SKT-27 exhibited the maximum nucleotide sequence identity (98.1%) with those of South African bovine strain 1603 (G6P[5]) and Thai bovine-like porcine strain P343 (G10P[5]) (Fig 2), and comparable identities (97.8–97.9%) with South African bovine strains (MRC-DPRU3010b (G6P[5]) and MRC-DPRU3005 (G6P[11])) and Slovene bovine strain SI-B17 (G6P[11]) [57]. On phylogenetic analysis, strain SKT-27 was shown to be clustered near these, and several bovine and bovine-like strains (Fig 13).

In the present study, we analyzed the whole genome of a Thai strain, SKT-27, with the G6P[14] genotypes (RVA/Human-wt/THA/SKT-27/2012/G6P[14]) from a child with acute gastroenteritis. Strain SKT-27 showed a unique genotype constellation: G6-P[14]-I2-R2-C2-M2-A3-N2-T6-E2-H3. The non-G/P genotype constellation of this strain (I2-R2-C2-M2-A3-N2-T6-E2-H3) is commonly shared with RVA strains from artiodactyls such as cattle. Phylogenetic analysis revealed that nine of the 11 genes of strain SKT-27 (VP7, VP4, VP6, VP2-3, NSP1, and NSP3-5) appeared to be of artiodactyl (likely bovine) origin. On the other hand, the remaining VP1 and NSP2 genes of strain SKT-27 were assumed to be of human origin. However, the exact origins of the VP1 and NSP2 genes of strain SKT-27 could not be ascertained due to a lack of a sufficient number of representative strains as references. Therefore, we could not determine if the suspected ancestral G6P[14] strain of strain SKT-27 had human-like VP1 and NSP2 genes in its genome before it was transmitted from cattle to humans. In any case, strain SKT-27 was assumed to be derived through interspecies transmission and/or reassortment events involving bovine and human RVA strains.

Notably, the VP7 gene of strain SKT-27 was located in G6 lineage-5, away from the clusters comprising other G6P[14] strains in G6 lineages-2/6. This could support the hypothesis that distinct human G6P[14] strains are more likely the result of individual interspecies transmissions from artiodactyls rather than the result of human-to-human transmission after interspecies transmission [18, 20]. To our knowledge, this is the first description of full genome-based characterization of human G6P[14] strains that have emerged in Southeast Asia.

Our findings add to the increasing evidence supporting animal-to-human interspecies transmission and reassortment events. The bovine origin of strain SKT-27 also suggests interspecies transmission due to the close proximity of humans to livestock, especially in developing countries in Asia where there is intimate contact between humans and livestock such as cattle. Because G6P[14] strains have been able to cause constant human infections worldwide, continuing RVA surveillance of this G-P combination in the human population may be required. Simultaneous monitoring of RVA strains in humans and animals is also essential for a better understanding of RVA ecology. Furthermore, whole genome-based analyses are essential to understand the evolutionary dynamics of novel RVA strains such as G6P[14] strains.
